# A Review of Biodegradable Natural Polymer-Based Nanoparticles for Drug Delivery Applications

**DOI:** 10.3390/nano10101970

**Published:** 2020-10-05

**Authors:** Humaira Idrees, Syed Zohaib Javaid Zaidi, Aneela Sabir, Rafi Ullah Khan, Xunli Zhang, Sammer-ul Hassan

**Affiliations:** 1 Department of Polymer Engineering and Technology, University of the Punjab, Lahore 54590, Pakistan; Aneela.pet.ceet@pu.edu.pk (A.S.); Rkhan.icet@pu.edu.pk (R.U.K.); 2 Institute of Chemical Engineering and Technology, University of the Punjab, Lahore 54000, Punjab, Pakistan; 3 Mechanical Engineering, Faculty of Engineering and Physical Sciences, University of Southampton, Southampton SO17 1BJ, UK; XL.Zhang@soton.ac.uk

**Keywords:** polymers, biodegradable, nanoparticles, drug delivery, pharmaceutical

## Abstract

Biodegradable natural polymers have been investigated extensively as the best choice for encapsulation and delivery of drugs. The research has attracted remarkable attention in the pharmaceutical industry. The shortcomings of conventional dosage systems, along with modified and targeted drug delivery methods, are addressed by using polymers with improved bioavailability, biocompatibility, and lower toxicity. Therefore, nanomedicines are now considered to be an innovative type of medication. This review critically examines the use of natural biodegradable polymers and their drug delivery systems for local or targeted and controlled/sustained drug release against fatal diseases.

## 1. Introduction

Enormous amounts of research and exploration of disorders and diseases have helped us to achieve an appropriate dosage system to stabilize a patient’s health [1,2]. Conventional drug delivery systems such as salting-out (salting-out agent required), supercritical fluid technology (capillary nozzle and a supercritical fluid required), dialysis (capillary nozzle and a supercritical fluid required), solvent evaporation (surfactant required), and nanoprecipitation (non-solvent for the polymer required) are also used [1]. Moreover, conventional drug delivery systems have several deficiencies such as reduced patient compliance, shorter half-life of drugs, and high peak, etc. There is, thus, increased interest amongst scientists in developing beneficial methods to improve drug delivery systems as time passes.

**Scheme 1:**
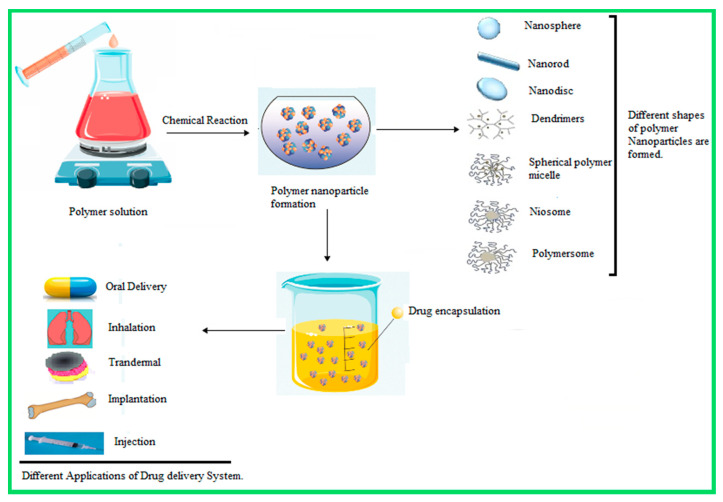
Representation of the general method of synthesis/preparation of nanoparticles.

A hundred years ago, Paul Ehrlich proposed the idea of tiny-drug loaded magic bullets. Later, the concept of a submicron drug delivery system was conceived by Kumar and Banker in 1996. Among these carriers, liposomes and micro/nanoparticles have been the most widely considered. Liposomes present a few technological limitations, such as poor stability, poor reproducibility, and low drug encapsulation efficiency. This technology is suitable for low molecular weight drugs. Hence, the polymeric nanoparticle drug delivery system has been proposed as an effective alternative drug delivery system to the conventional system [3]. Scheme 1 illustrates the general preparation methods for nanoparticles and their applications for drug delivery systems.

Brachais et al., in 1998, prepared a solid dispersion method to synthesize an orally-controlled drug release system. They used a biodegradable hydrophobic matrix, poly (methyl glyoxylate), and a water-soluble drug, metoprolol. Jeong et al., in 1999, studied the star-shaped block copolymers using bio-degradable polyethylene oxide and poly (L-lactic acid), in which copolymers exhibit reversible sol-gel transition [4]. Avnesh Kumari et al., in 2010, reviewed biodegradable nanoparticles such as chitosan, gelatin, poly(lactic-co-glycolic acid), Polycaprolactone, PLA(Poly Lactic acid), and gelatin as having better encapsulation properties for drug release [5]. In 2014, Carlotta Marianecci et al. presented a review on surfactant vesicles, which generated interest among the scientific community in the last decades. They studied how niosomes, which are self-assembled vesicular nanocarriers for the drug delivery system, overcome the side effects of liposomes due to their less-toxic effect, are stable, and have a low cost [6]. Jeong et al., in 1999, studied star-shaped block copolymers using bio-degradable polyethylene oxide and poly (L-lactic acid); these copolymers exhibit reversible sol-gel transition.

Therefore, exponential growth in the development of modified drug delivery systems is essential for dosage form improvement. Modified drug delivery systems have been considered to transport active agents in higher demand due to their delivery process, programmed target-specificity, cellular uptake, clearance, toxicity, metabolism, pharmacokinetics, excretion, greater half-life in terms of repeated administration of drugs and improved patient health [7]. The successful drug delivery systems are designed to increase the efficiency of the drug in the body by using external or internal stimuli, and nanocarrier features are modified according to the physicochemical properties of drugs [2]. Indeed, drugs essentially require highly effective, controlled release along with biocompatible encapsulation to increase patient compliance [2].

Due to the rapid increase in research in the field of polymer science, the structural backbone for the development of novel and modified drug delivery systems is considered [8]. Primarily, non-biodegradable polymeric nanoparticles, such as polymethylmethacrylate, polyacrylamide, and polystyrene, have been used for drug delivery systems; however, a huge level of toxicity and detrimental health consequences from non-biodegradable polymers have been observed. Therefore, biocompatible polymers, due to their immense properties and growth, are under discussion within the scientific community for in vivo and in vitro diagnosis and treatment of diseases [8,9,10].

Polymeric nanostructured materials (PNMs) have played a vital role in therapeutic diagnosis and treatment of diseases [11,12,13,14]. Through the development of PNMs as new biomaterials, significant improvement in the quality of healthcare can be achieved, due to the better accuracy and reliability in diagnostics, more effective targeting of therapeutic agents, and improved usability of scaffolds for tissue engineering and regenerative medicines [15,16,17]. PNMs, including micelles, polymerases, nanoparticles, nanocapsules, nanogels, nanofibers, dendrimers, brush polymers, and nanocomposites can be prepared for delivery via a variety of pathways. Their properties, such as stability, size, shape, surface charge, surface chemistry, mechanical strength, and porosity can be tailored toward specific functionalities that are required to meet the needs of the targeted biomedical application. As a result, the development of biomedical PNMs has attracted plenty of research in the field, and a vast number of recent publications can now be found in the literature.

**Figure 1:**
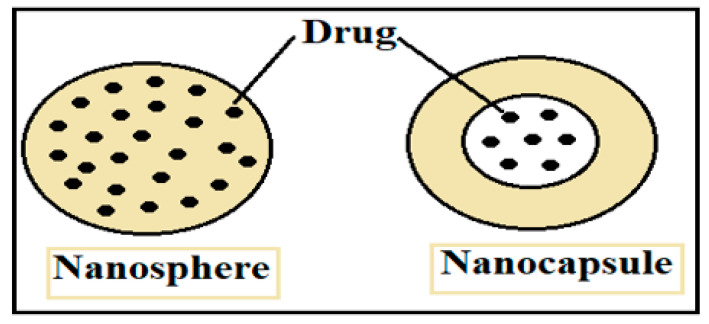
Schematic of nanomedicines: nanospheres and nanocapsules.

An amalgamation of nanoparticles with polymer science has led to a new direction in the field of biomedical engineering, packaging, food processing, tissue engineering and improved treatment for water-insoluble and soluble drug delivery systems. Nanoparticles are characterized by a particle size range of 1 to 100 nm. ‘Nano’ is derived from the Greek word ‘Nanos’ which means dwarf [9,10,11,12,13,14,15,16,17,18]. The basis of the morphology, chemical and physical properties of nanoparticles relate to the different derivative materials such as ceramic, liquid, metal, semiconductors, and carbon-based nanoparticles, which may be biodegradable or non-biodegradable [19]. Nanoparticles or smart polymers are under consideration due to their advantages, including a greater surface to charge ratio, ease of characterization and ease of synthesize, the fact that they are reproducible, stable after administration, non-immunogenic, and have significant absorption properties. These unique features make them of huge interest as carriers for drugs and inexpensive formulation [20]. The effective therapeutic transformation from a macro- to a nano-drug delivery system relates to the controlled release of a drug on a target as compared to the less targeted release of conventional drug delivery systems [21]. Therefore, depending upon the method of synthesis, nanomedicines can be one of two types, i.e., nanospheres which encapsulate drugs into a smart polymeric shell (nanopolymer shell) and nanocapsules in which drugs are dispersed into the polymeric matrix [22,23]. The representation of both types of nanomedicines is shown in Figure 1. The synthesis of a nano-drug delivery system depends upon the targeted part of the body and organ. The biocompatibility and degradability of nanomedicines must be considered as they carry DNA, drugs, and proteins to the targeted area [24]. The size and surface-to-charge ratio of polymer nanoparticles play a significant role in maintaining systematic flow across the cell membrane [25,26]. The efficiency of the drugs depends upon the properties of the polymer, nanoparticles, solvent, and encapsulation method.

**Figure 2:**
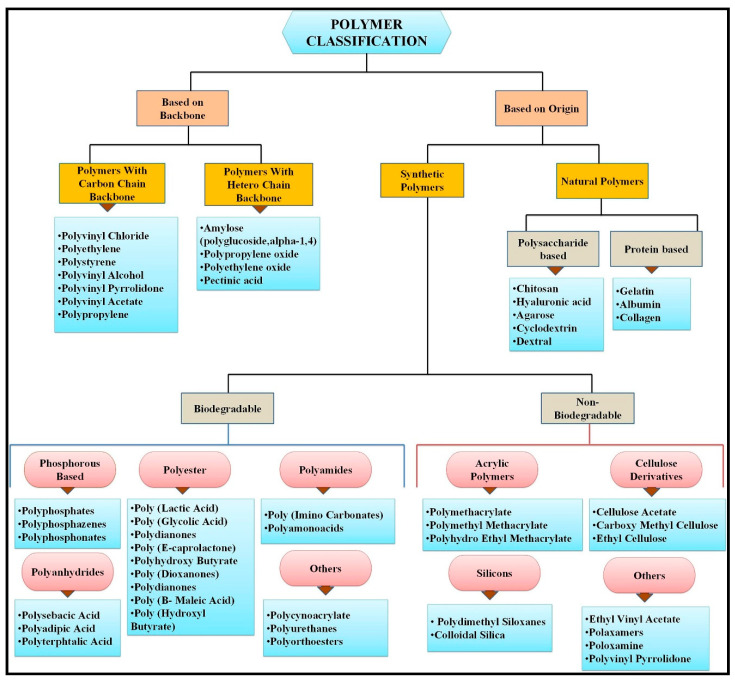
Schematic of polymer classification. Reference is taken from [35]. Copyright ©2019, Elsevier.

Smart polymers or nanoparticles play an essential role in the treatment of chronic diseases such as cancer, diabetes, cardiovascular and neurodegenerative disorders [27,28]. Various natural and synthetic polymers play a significant role in a targeted drug delivery system [29,30]. Some natural and synthetic polymeric materials have easy accessibility, biocompatibility, bio-decomposition properties, and easy modification. Reactive groups, such as amino, hydroxyl and carboxylic groups present on polymers, easily interact with other synthesized materials, thus endowing new modified hybrid materials with improved physical and chemical properties [31,32,33]. There are various types of natural polymers such as proteins, polysaccharides, peptides, collagen, albumin, gelatin, chitosan, alginate, fibroin, and synthetic polymers such as polylactic acid (PLA) and polyglycolic acid (PGA) [34]. Synthetic and natural polymers have their disadvantages and advantages. Biocompatible polymers are mostly preferred because they are economical, easily prepared, and extremely stable in the biological fluid; they show better proliferation, adhesion, and target usage with high efficiency [34]. Nanomaterials usually consist of carbon-based materials such as fullerenes, carbon dots, nanodiamonds, nano-foams, carbon nanotubes, and polymers. Inorganic nanoparticles are metals such as gold, silver and metal oxides (cerium oxide, iron oxide, silicon dioxide, titanium oxide), semiconductors and metal nanoparticles [33]. Figure 2 shows the schematic of the polymer classification.

In general, the aim and specificity of the present review are highly important for some natural, biodegradable and biocompatible polymers. The present review covers some popular polymers such as chitosan, albumin, alginate, hydroxyapatite, and hyaluronic acid currently used for drug delivery systems. Furthermore, it provides information about the functionalization of the above-mentioned polymers to enhance their properties and develop an effective drug release system [36].

## 2. Biodegradable and Non-Biodegradable Polymer Nanomaterials (PNM): General Properties

### 2.1. Biodegradable Polymer Properties

Biodegradable polymers undergo degradation, non-enzymatically and enzymatically and generate a harmless, biocompatible by-product. Biodegradable polymers have a notable emphasis on the chemistry in the scheme of new molecules in targeted drug delivery applications. The use of biocompatible polymers reduces the side effects of a given drug. Biodegradable biomaterials have no constant inflammatory effect, good permeability, and good therapeutic properties. The performance of biodegradable polymers depends upon the following aspects:

(1) In situ administration of formulations
(2) On-demand delivery of the molecular targeted agent
(3) The fact that a targeted agent can be combined with radiotherapy and immunotherapy
(4) The use of FDA approved biodegradable polymers [35]

### 2.2. Non-Biodegradable Polymer Properties

Clinically non-biodegradable polymers are used for local injection of antibodies. Mostly non-biodegradable polymers such as acrylic polymers, cellulose derivatives, silicons, etc. are mentioned in Figure 2 in the introduction section. Polymethyl methacrylate (PMMA) is an acrylic-based non-biodegradable polymer that is mostly used for implantation as bone cement or in the form of PMMA beads. While widely used, PMMA has many disadvantages: it is a substrate for bacterial colonization, has low biocompatibility, is non-biodegradable in bead form, the release of antibiotic decreases with time, it is not heat resistant, it has fewer antibiotic elimination properties, delivery of the antibiotic, it has a variable surface area which results in uneven release rates. Due to the disadvantages of non-biodegradable polymers, scientists are interested in developing biodegradable, biocompatible polymer synthesis for a drug delivery system [37]. The main objective of this review is to study the role of five biodegradable polymers in a drug delivery system.

## 3. Chitosan-Based Nanoparticles

**Figure 3:**
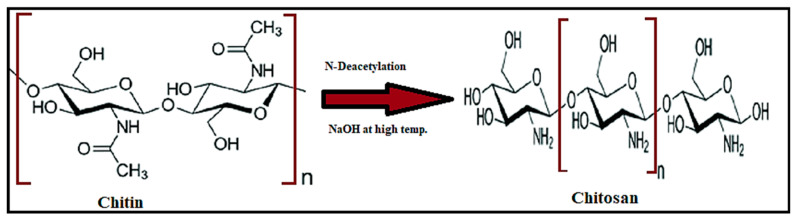
Structure of chitosan.

Chitosan is a polysaccharide, with 2-deoxy-2-(acetylamino) glucose units bonded by 1,4-glycosidic linkages; it is prepared by partial N-deacetylation of chitin (Figure 3). Chitin has good mechanical strength, is biocompatible, bioactive and biodegradable, but has limited utilization due to low solubility. Therefore, it is converted into chitosan by deacetylation in the presence of hydroxide at high temperatures [38]. Chitin is extracted from marine organisms such as lobsters and molluscs, and from crab shells, insects, yeast, and fungi. A long polymeric chain is composed of glycosidic linkages. Chitosan is insoluble in sulfuric acid and phosphoric acid but soluble in organic solutions with a pH lower than 6.5, such as acetic acid, citric acid, and tartaric acid. Due to its solubility, chitosan is present in films, hydrogels, pastes, nanoparticles, and nanofilms [39,40]. Chitosan has a greater degree of deacetylation and molecular weight; the variation in the size of a particle and aggregation is dependent upon the degree of deacetylation and molecular weight [3]. Recently, chitosan and its derivatives have been considered as the best vehicle in the pharmaceutical field due to their biocompatibility, and their non-carcinogenic, non-toxic, antibacterial properties. Chitosan offers a large range of options for industries and scientists for the generation of modified and novel drug delivery systems. Chitosan acts as an auxiliary agent in the therapeutic application for tissue engineering, wound dressing, and sliming. Protonation of the amino group in an acidic medium results in cation formation. Chitosan exhibits unique behavior because of its cationic nature [38,41]. Modification of chitosan and the stability of drugs delivered using chitosan decrease the adverse effects of diseases and increase the biocompatibility of drugs for various diseases [42].

Chitosan nanoparticles widely act as a potential carrier for therapeutic applications. The cationic nature of chitosan is useful for the development of a drug delivery system. One of the major benefits of chitosan nanoparticles is their rapid uptake across the cell membrane due to the presence of amine groups. Complexation of chitosan with anionic charged polymers results in an interesting gelation property [43]. Methods generally applied for the preparation of chitosan nanoparticles include the microemulsion method, ionic gelation, and micro-emulsion solvent diffusion method [3,38]. In the microemulsion method, chitosan nanoparticles are prepared, and micellar droplets are cross-linked in the presence of glutaraldehyde. Surfactant (hexane mixture) is added to the chitosan acetic/glutaraldehyde solution. Surfactant helps in the formation of chitosan nanoparticles smaller than 100 nm under continuous stirring to complete cross-linking between the amine group of chitosan with glutaraldehyde. By applying a low pressure, the excess organic solvent is removed. The excess organic solvent used, the complexity of the washing process, and the amount of time this method takes is its main drawback [3,44]. Chitosan nanoparticles prepared using the inotropic gelation method depend upon electrostatic interaction between the amino group of chitosan and the polyanions group such as triphosphate in an aqueous medium [45,46,47,48]. Firstly, the chitosan is dissolved in acetic acid in the presence of stabilizers such as poloxamer. So, nanoparticles are formed under continuous stirring. The size of the nanoparticle depends upon the ratio of chitosan-to-stabilizer [3]. The emulsion solvent diffusion method is based on the addition of an organic phase into a chitosan solution containing a stabilizer at a higher temperature and pressure with constant stirring. Nanoparticles are formed by the addition of water to an organic solvent. This method of nanoparticle formation is better for hydrophobic-based drug delivery systems. This method also has some drawbacks, such as greater shear force and harsh process conditions [49]. Chitosan is one of the most extensively studied biopolymers because chitosan possesses some ideal properties for polymeric carriers for nanoparticles. The properties are given in Table 1.

**Figure 4:**
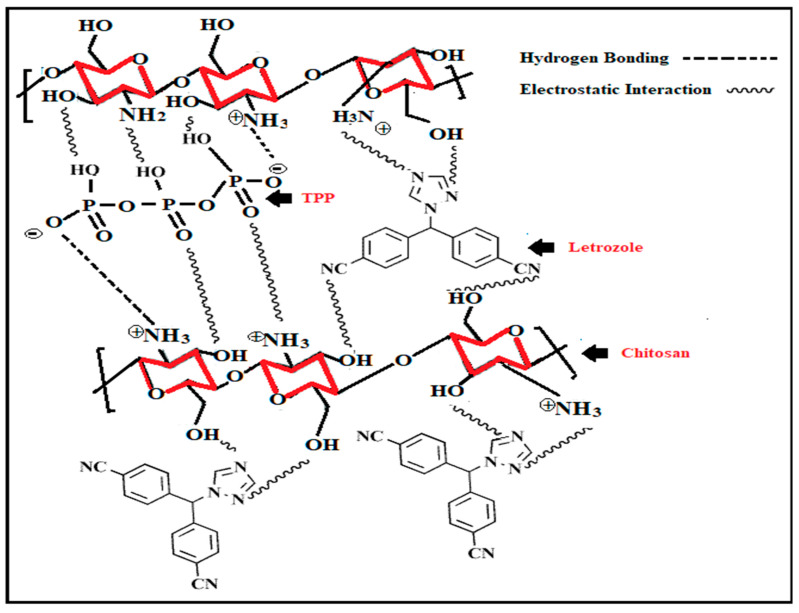
Shows the formation of chitosan nanoparticles modified with tripolyphosphate (TPP)-loaded letrozole [52]. Copyright ©2017, Elsevier.

Current studies have shown a massive variation of applications and novel modifications of chitosan, thereby increasing its overall value. Drug encapsulation using chitosan with tripolyphosphate was first reported by Bodmeier et al. [50,51] who used an inotropic gelation process, which results in the formation of chitosan nanoparticles. Chitosan (100–150 nm) nanoparticles were loaded with Rosuvastatin drug encapsulated in polyvinyl alcohol (PVA)/Sodium alginate (SA) core using an ionic gelation method. The release behavior of the drug was observed within 24 h, and chitosan particles delivered significant results in the drug release process. This biocompatible and biodegradable drug delivery system was a suitable choice for replacing the various doses of the drug Rosuvastatin [51]. In 1998, Alonso et al. synthesized the chitosan nanoparticle with the development of inter-and intra-molecular interaction between the chitosan amino group and tripolyphosphate (TPP). This method produced a high yield of chitosan nanoparticles [43]. Thandapani Gomathi et al., in 2017, synthesized chitosan nanoparticles with TPP-loaded drugs with letrozole (LTZ) for cancer treatment. Characterization techniques such as SEM, TEM, FTIR, XRD, and TGA results showed the optimum results. Additionally, the prepared formulation was evaluated in vitro to determine its biodegradability, hemocompatibility, and serum stability. The preliminary studies supported the assertion that chitosan nanoparticle synthesis had biocompatibility and hemocompatible applications, and could act as an essential pharmaceutical excipient for letrozole [52]. Synthesis techniques for chitosan nanoparticles are given in Figure 4.

The use of chitosan nanoparticles was reported by Wang et al., in 2017, for insulin delivery [53]. Zhang et al. modified chitosan by using heptamethine and folate for photodynamic treatment and tumor-imaging [54]. Kamel et al. modified chitosan by encapsulating oregano and cinnamon within chitosan in combination with 5-fluorouracil as an effective agent for tumors [55]. Lin et al. studied chitosan derivatives and found that chitosan-based nanocarriers used for co-delivery of genes and drugs were redox responsive [56]. In 2017, Gu et al. reported antibody modified nanoparticles. The delivery of drugs to the brain has always been a challenging task because the blood–brain barrier does not allow quick cellular uptake to the brain [57]. Auspicious work has been done by Par et al. for cancer treatment; the authors used glycol-modified chitosan nanoparticles for the effective release of doxorubicin [58]. In 2018, Belbekhouche et al. synthesized chitosan/polyacrylic modified nanoparticles for drug delivery systems [59]. A considerable amount of experimental work has been reported by Bernkop-Schnürch et al. [41]. The authors studied the efficiency of chitosan for different drug delivery systems: oral, gastric (for cancer treatment), nasal, buccal, intravesical, ocular, and, additionally, systems for vaccine delivery. The properties of chitosan nanoparticles vary when coupled with a hydrophilic polymer. This modified form of chitosan has shown novel susceptibility for delivering protein and interaction with the biological surface [43]. Calvo et al. examined the remarkable properties of modified chitosan using the diblock copolymer of ethylene oxide and propylene oxide as a protein carrier. The introduction of a PEG coating on the surface of chitosan decreases its cationic surface charge and, remarkably, increases its biocompatibility [60].

In 2002, Shu and Zhu [61] studied the effect of electrostatic interaction on properties of chitosan ionically crosslinked with multivalent phosphates such as tripolyphosphate, phosphate and pyrophosphate. Chitosan cross-link ionically due to the greater negative charge on the surface of phosphates. The solution pH plays a vital role in electrostatic interaction between chitosan and multivalent phosphates. Pyrophosphate/chitosan exhibits greater interaction as compared to other tripolyphosphates and phosphates. Ionically cross-linked chitosan and tripolyphosphates displayed better surface charge to size ratio for particles and showed better association with vaccines, proteins, plasmids, peptides, and oligonucleotides [61]. Recently many issues related to cancer treatment, such as hematogenous metastasis, drug resistance and local reappearance have led to the failure of treatment methods [62]. The main problem related to cancer treatment is finding a drug delivery system that fits the requirements. Shafabakhsh et al. [38] reported a review of gastric cancer treatment using chitosan nanoparticles. The authors investigated the effect of the anticancer drug norcantharidin conjugated with carboxymethyl modified chitosan.

In comparison with the simple drug, the carboxymethyl/chitosan encapsulated the drug successfully and suppressed the migration and proliferation of gastric cancer cells. This study reported that carboxymethyl chitosan could increase gene expression and may provide a favorable drug delivery system for gastric cancer treatments [38,63]. Moreover, chitosan nanoparticles used for the treatment of H. pylori infection have been shown to improve the effect of amoxicillin; chitosan nanoparticles improve the release time of the drug by preventing them from enzymatic and acidic breakdown through bonding to the mucus barrier of the stomach and drug release into the mucus barrier, thus leading to greater efficacy at the infected site [64,65].

Øilo et al. [66] suggested dental coating by using modified chitosan with other alternative antibacterial agents. The formation of biofilms causes common dental diseases that involve microbes adhering to teeth or restorative materials. Microbial adhesion is followed by bacterial growth and colonization, resulting in the formation of a compact biofilm matrix [67]. This matrix protects the underlying bacteria from the action of antibiotics and host defense mechanisms. The biofilm formed on teeth, prostheses, or implant-anchored restorations contains aciduric organisms such as *Streptococcus mutans* (*S. mutans*) and *lactobacilli* that secrete acid causing enamel and dentin demineralization. Biofilm formation on dental implants can result in a severe infection leading to dental implant failure. The formation of biofilm is reduced by different antibacterial agents such as quaternary ammonium compounds [68], inorganic nanoparticles [69], or fluoride varnish with natural products [70], which are used in the dental materials. Dental varnishes containing fluoride with natural products such as chitosan are a practical approach. Newer techniques include the use of an antibacterial polymer coating drug delivery system to prevent bacterial growth on artificial tooth surfaces in other dental materials and dental composite kits, increasing the longevity of the dental restoration [71]. Examples of such antibacterial coatings include copolymers of acrylic acid, alkyl methacrylate, and polydimethylsiloxane copolymers [72], pectin coated liposomes, and carbopol [73].

## 4. Alginate-Based Nanoparticles

**Figure 5:**
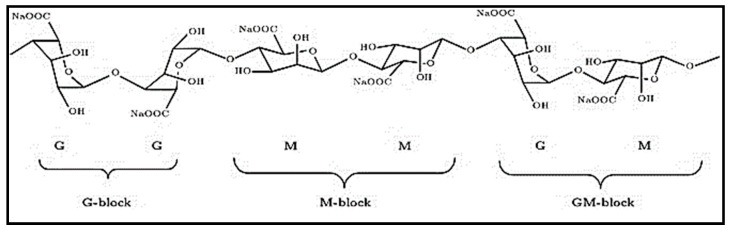
Types of alginate blocs: M = mannuronic acid; G = guluronic acid [75].

Alginates are a group of the most important biopolymers, also known as sodium-alginates, which are unbranched polysaccharide anionic polymers (Figure 5). Alginate demonstrates a wide range of potential applications in a polymeric drug delivery system, in the food industry in the form of additives based on electrostatic interaction. A modified form of alginates has introduced a greater level of properties in the biomedical and pharmaceutical industries [74]. Alginate is made up of unbranched polysaccharides extracted from brown seaweeds and soil bacteria. Alginic acid is the resulting product extracted from seaweed which is converted into sodium alginate, currently used in the pharmaceutical industry as an effective drug carrier. Alginic acid is a linear polymer consisting of L-guluronic acid and D-mannuronic acid; these are linearly arranged in the polymer chain. Alginates from various sources differ in their extents of blocks. Hydration of alginic acid leads to the synthesis of a high-viscosity “acid gel” due to intermolecular binding. Due to gelation, water molecules are enclosed inside the alginate matrix but are still free to migrate, which has a great application in drug encapsulation and cell immobilization [74].

Alginate is a perfect polymer for chemical functionalization, due to its free carboxyl and hydroxyl groups among the backbone. Properties such as hydrophobicity and solubility, and physicochemical and biological characteristics are enhanced due to the formation of alginate derivatives based on hydroxyl and carboxylic groups. Many physical and chemical methods, such as polymer blending, grafting copolymerization with hydrophilic vinyl monomers, and compounding with other functional components, can be used to modify sodium alginate [76]. Divalent cations act as cross-linkers between the functional groups of alginate chains. Polyvalent cations such as Ca^2+^, Sr^2+^, or Ba^2+^ are responsible for intrachain and interchain cross-linking of alginate, forming insoluble alginate with the anionic polymer. Calcium is the main cation used because it is considered to be simply accessible, clinically safe, and cost-effective. The reaction of sodium alginate and calcium ion consists of a simple cross-linking process in which sodium-alginate is converted into calcium alginate [77].

2Na(Alginate) + Ca^2^^+^ → Ca(Alginate)_2_ + 2Na^+^

Wandrey et al. [77] reported that, in the development of high mechanical strength and greater permeability, G alginate showed advantageous properties, and for additional properties, M alginate was suggested. Amphiphilic alginate is a current choice for drug delivery systems, and it shows properties such as low toxicity, good biocompatibility, mechanical stiffness, binding and release of drugs upon modification; it also reduces side effects and increases affinity with drugs [78,79]. Alginate-based smart polymers respond to pH [80], temperature [81], light [82], enzymes and magnetic field [81,83]. Most of the drug carriers are synthesized on the basis of an ionic complexes of alginate or its sulfate derivatives with a cationic macromolecule such as peptides or proteins. Wu et al. [84] reported on a nano-sized drug carrier for chemotherapeutic applications based on inorganic/organic hybrid alginate/CaCO_3_ using the co-precipitation method under optimal conditions in the presence of an aqueous solution. A hydrophobic drug (paclitaxel, PTX) and hydrophilic drug (doxorubicin hydrochloride, DOX) were co-encapsulated in the alginate/CaCO_3_ hybrid nanoparticles. Different characterization techniques were used to observe the behavior of a simple nanoparticle-encapsulated drug and a co-encapsulated one. The drug-loaded with modified nanoparticles showed greater cellular uptake and an enhanced inhibitory effect. These results showed that alginate/CaCO_3_ hybrid nanoparticles have beneficial applications for the co-delivery of drugs with altered physicochemical properties. In another study, Jahanban-Esfahlan et al. [84] reported on an effective and drug release system for tumor treatment. They developed magnetic natural hydrogel based on alginate (Alg), Fe_3_O_4_ magnetic, and gelatin (Gel), nanoparticles (MNPs). Firstly, alginate was partially oxidized, then a shift-base condensation reaction was used to develop alginate-gel. Secondly, using a co-precipitation method, Fe_3_O_4_ nanoparticles were introduced in prepared alginate-gel. Characterization showed that this method synthesized hydrogel without any micro-phase separation. The attained Alg-Gel/Fe_3_O_4_ was loaded with doxorubicin hydrochloride (DOX), its drug loading, encapsulation properties and anticancer movement, were examined against Hela cells. The presence of carboxylic acid in the drug delivery system (synthesized Alg-Gel/Fe_3_O_4_-DOX) showed pH-dependent drug release. This modified form of alginate with magnetic nanoparticles showed promising results for a smart drug delivery system.

Moreover, Gao et al. [79] presented hydrophobic drug-based self-assembled micelles, a dual-stimuli responsive drug delivery system for hydrophobic drugs. Alginate was modified with dodecyl glycidyl as a hydrophobic group and was able to form self-assembled micelles in the aqueous solution above the critical micelle concentration. Doxorubicin (DOX) was used as an exemplary drug and successfully loaded in HMA (hydrophobic modified alginate) micelles. The enzyme and pH-stimuli release behavior of DOX from DOX-HMA micelles was such that the release of DOX was enhanced in an acidic medium. The release of drugs in the presence of a catalyst named an Alpha-Lfucosidase was effectively increased. Zhang et al. [85] synthesized graphene functionalized alginate (GO-ALG) for colon cancer treatment. This modification opened the way for many other therapeutic applications. The main issue linked with colon cancer is liver metastasis. Conventional drug delivery systems have many problems, such as failure to control the drug release ratio, poor stability, wrong targeting, and exposure to the microenvironment. This study reports the synthesis of graphene oxide (GO)-functionalized, sodium alginate (ALG) colon-targeting drug delivery system, with 5-fluorouracil (5-FU) used as the sample anticancer drug. The results showed that modified GOALG/5-FU expressively stopped tumor growth and liver metastasis and increased the life span of mice. This research opened a new route for the treatment of colon cancer liver metastasis. Nazemi et al. [86] investigated the ionic complexation of various configurations of alginate and its sulfated derivative with tetracycline hydrochloride (TCH). Remarkably, the functionalization of alginate with sulfate groups resulted in drug-polymer complex formation. Mannuronate-enriched alginate complex with TCH trapped the greater quantity of the drug. The results showed that this modification of alginate with TCH comes out as a favorable biomaterial for a cationic drug delivery system. Hügl et al. [87] entrapped the neurotrophic factor producing cell in an alginate polymer matrix. The application processes were tested for their potential in an artificial human cochlea model. Since the methods potentially affect the electrode implant capacity, the coating stability and insertion forces were analyzed on custom-made electrode arrays. Both inoculation of the alginate-cell solution into the model and a manual dip coating of electrode arrays with successive insertion into the model were promising. The filling of the model with a non-cross-linked alginate-cell solution improved the insertion forces. A good stability of the coating was examined after the first supplement. Both application schemes are a promising choice for cell-induced drug delivery to the inner ear, but an alginate-cell coating of electrodes has great potential with a reduction of insertion forces.

## 5. Albumin-Based Nanoparticles

**Figure 6:**
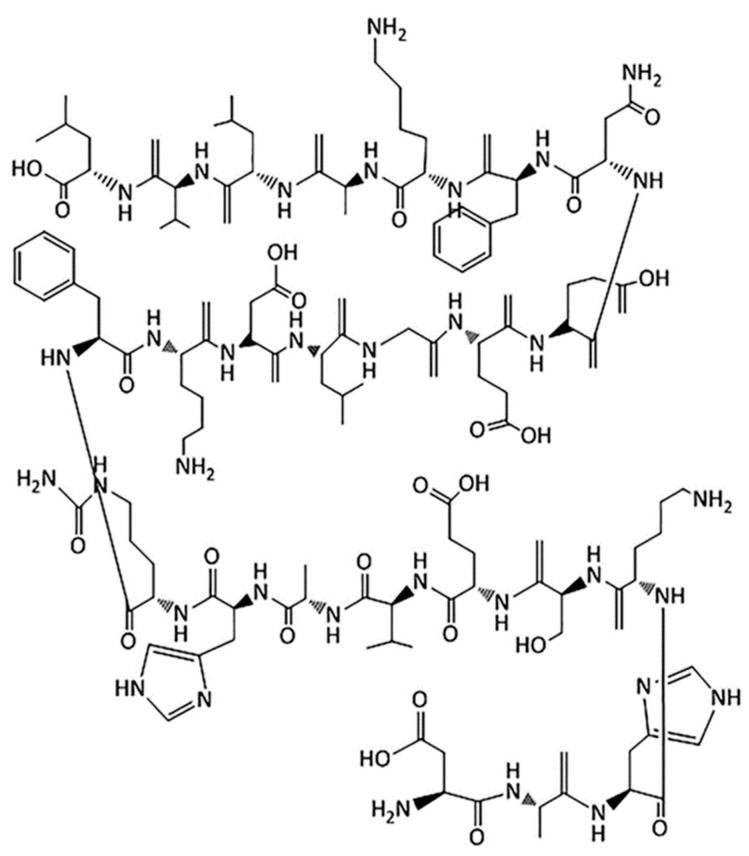
Structure of albumin [8]. Note that the oxygen atoms of almost all the carboxyl groups of amino acids are not shown in the structure. Copyright ©2019, Elsevier.

Albumin is a natural, water-soluble globular protein and attractive macromolecular carrier which has a biodegradable property. Albumin is non-immunogenic, non-toxic, non-antigenic and biocompatible [88,89]. A large number of drugs can be incorporated into the nanoparticle-matrix because albumin molecules have different binding sites [90]. Albumin structure is shown in Figure 6.

**Figure 7:**
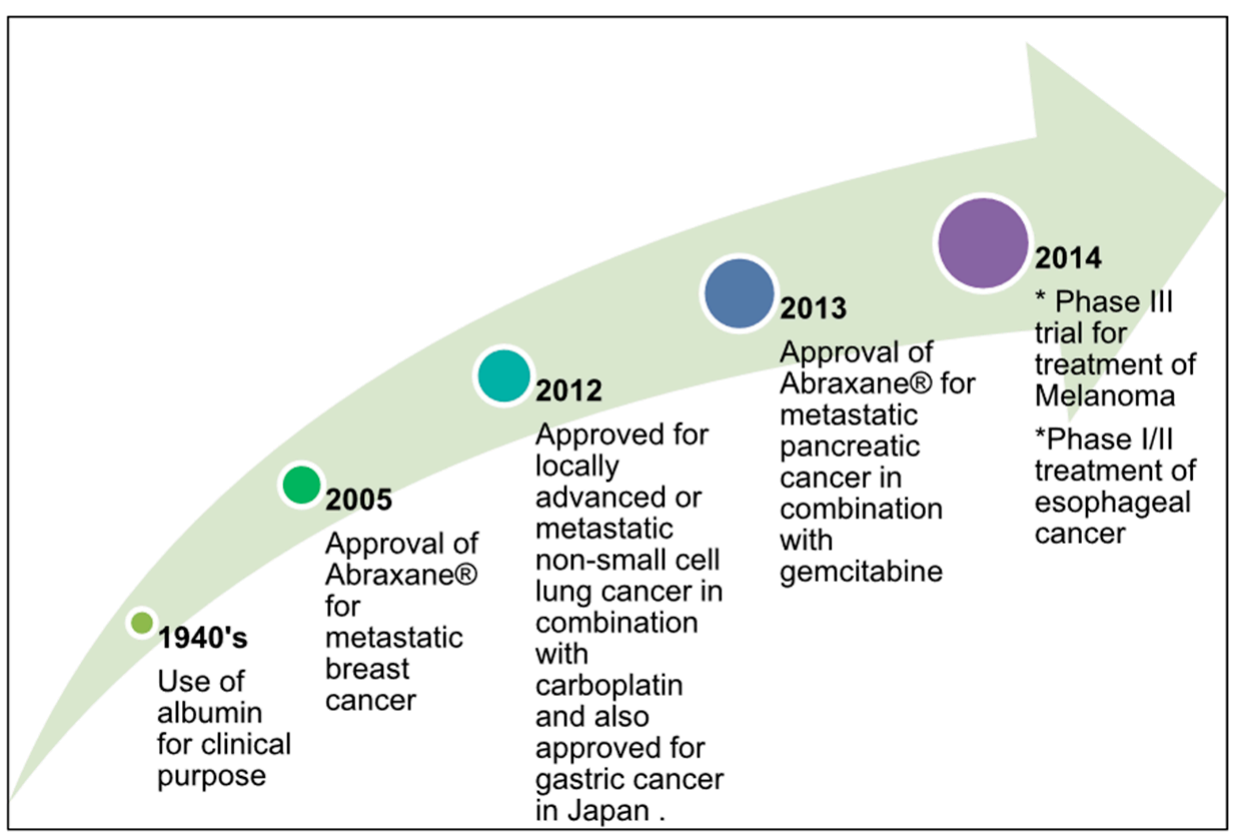
The successful use of albumin and an albumin nanoparticle (Abraxane) for cancer treatment [89]. Copyright ©2020, Elsevier.

Albumin-based nanoparticles allow electrostatic interaction of cationic and anionic charge; drugs show non-covalent and covalent interaction with albumin nanoparticles. Figure 7 demonstrates the successful use of albumin and albumin nanoparticles for cancer treatment [89]. The presence of primary amino acid groups in albumin, such as lysine, indicates a vital role in cross-linking [91]. Albumin is prepared by controlled desolvation, coacervation, and emulsion formation. Commercially available forms of albumin are ovalbumin (egg white), human serum albumin, albumin extracted from soybeans, albumin present in bovine serum capsules, grains, and milk [92], where egg albumin has a molecular weight of up to 47,000 Da.

Furthermore, ovalbumin is non-toxic, sensitive to temperature or pH, and economical; it gives effective results in food matrix design, stabilization of foams and emulsions, and a is a good candidate for sustainable drug release [93]. A bovine serum capsule has a size of up to 69,323 Da, and due to its non-toxic behaviour, and ligand binding property it is extensively used for drug delivery applications [94]. Human serum albumin has a molecular weight of 66,500 Da and peripheral uptake, non-toxicity and biodegradability properties make it beneficial for pharmaceutical applications [95]. Albumin nanoparticles have several useful properties such as easy incorporation of various drugs and the ability to bind with proteins, due to presence of carboxylic and amino group on nanoparticle surface [96].

Modification of albumin with PEG has not only improved the blood circulation but also provides a gateway in the pharmaceutical industry for cancer treatment drugs [97]. Albumin nanoparticles show a better affinity for cancer treatment drugs such as doxorubicin, curcumin, Abraxane, and tacrolimus [98]. Kim et al. [98] prepared HSA nanoparticles loaded with curcumin; these nanoparticles show higher solubility. Dreis et al. [99] have developed a system for the preparation of doxorubicin-loaded HSA nanoparticles. Using doxorubicin-loaded HSA nanoparticles, the toxic effect of anticancer drugs was reduced, and the multidrug resistance issue was resolved. Joshi et al. [89] discussed nanocarriers for pulmonary cancer. Surface modification of a nanoparticle helps in the effective movement of nanoparticles across the mucus layer. Surface functionalization of nanoparticles was performed using various methods such as adsorption, conjugation, and surface coating. Here, the surface of albumin modified with a neutral molecule polyethylene glycol enabled the movement through the mucus layer of the respiratory tract [89]. Surface modification based upon the required application is possible due to many reactive groups on the surface of albumin. Iwao et al. [100] presented a strategy for a site-specific drug delivery system for the cure of ulcerative colitis (UC). The authors prepared modified human serum albumin (HSA), and myeloperoxidase (MPO) and prepared nanoparticles (HSA NPs) conjugated with 5-aminosalicylic acid (5-ASA). The specific contact between 5-ASAHSANPs and MPO was examined using quartz crystal microbalance analysis.

Furthermore, Siri et al. [101] presented the effect of an albumin nanoparticle structure with its function as a drug release system. In this study, albumin nanoparticles were irradiated with gamma rays (cross-linker) by using the desolvation method. This method causes albumin nanoparticles to generate new hydrophobic pockets which make it a sound drug delivery system. The hydrophobic drug, Emodin, was used as a sample to check the release behavior. The formation of nanoparticle pockets enhanced the encapsulation property of the system. Stein et al. [102] studied the preparation of stable mTHPC-albumin nanoparticles using nanoparticle albumin-bound (nab)-technology to develop a system for drugs that are not very water-soluble. In this study, the advantages of nanotechnology and albumin with the ability of high tumor enrichment and the selective light initiation of the photosensitizer Temoporfin (mTHPC) were associated with a new delivery system for reliable tumor treatment. The nanoparticles were characterized according to size distribution and particle size, and the effect of this method on the nanoparticles as well as mTHPC stability was studied. Table 2 shows polymer/polymer modified chitosan-, alginate-, and albumin-based nano polymers for drug delivery systems.

## 6. Hydroxyapatite-Based Nanoparticles

Hydroxyapatite (HAp) has great applications in the biomedical field and is considered the best option in the pharmaceutical field due to its excellent bioactivity and biocompatibility. Hydroxyapatite is derived from the mineral compounds of human bones, teeth, and hard tissues. The basic units of HAp are calcium and phosphates (CaP) characterized as M1_4_M2_6_(PO_4_)6(OH)_2_, in which M1 and M2 are two crystallographic arrangements. The stability of CaP is directly related to the presence of water molecules during synthesis and the medium where it was applied [103].

Furthermore, HAp shows good mechanical strength, a porous structure, osteointegration, and osteoconductive properties. HAp can be used as an implant material due to its granular particles, porous structures, load-bearing ability and excellent biocompatibility. The combination of HAp with phosphates, such as calcium pyrophosphate and β-tricalcium phosphate (β-TCP), means that this material has a vast number of properties. The use of hydroxyapatite in the implantation, free layer of fibrous tissue composed by carbonated apatite is generated on its surface, which helps in the binding of the implant to the living bone through an osteoconduction mechanism. HAp prevents any toxicity effect during implantation, and its porous structure gives excellent diffusion properties. HAp is used in tissue engineering as a scaffold, in dental enamel repair, in medicine, for cancer cell treatment and as a bone cement [104,105]. A recent study investigated that HAp shows strong bonding due to its porous structure [106]. HAp powder was synthsized using a hydrothermal method with the use of calcium nitrate [Ca(NO_3_)_2_.4H_2_O] for calcium and potassium dihydrogen phosphate [(NH_4_)_2_HPO_4_] and phosphorous, and it was used as a precursor. The use of HAp derivatives is in high demand in the field of orthopedics. This is undoubtedly beneficial for both commercialization and research purposes; it means that the use of composites is studied and experimented with, enhancing the performance of the previous defective bones. Li et al. [107] successfully synthesized a core–shell nanocarrier (PAA–MHAPNs) based on a grafting method. This synthesized system showed excellent results; for example, it had improved loading in terms of the quantity of anticancer drug (doxorubicin hydrochloride), electrostatic properties, and promising for application in pH-sensitive drug release systems. The loading capacity increased up to 79% at low pH. The cytotoxicity analyses designated that the PAA–MHAPNs was biocompatible. Overall, the synthesized systems have great ability as drug nanocarriers for drug delivery, excellent biocompatibility, and pH-responsive features for future intracellular drug delivery. Venkatasubbu et al. [108] presented functionalized hydroxyapatite (HAp) with folic acid (FA) modified polyethylene glycol (PEG) for therapeutic applications. In this study, in vitro analysis of anticancer drug (paclitaxel) loading in modified HAp was performed. The authors studied the initial rapid release of the drug and then the sustained release, and presented a review of three types of hydroxyapatite-based nanoparticles: magnetic HAp, luminescent HAp, and immunomagnetic HAp for bioimaging applications. Various research work on the antimicrobial property of HAp nanoparticles was presented in this study. Additionally, the silver doping particle in HAp increases its antimicrobial property by cross-linking a silver nanoparticle with a thiol group of bacteria, and HAp shows good compatibility with bone marrow stem cells [109]. Table 3 shows the advantages, disadvantages, biomedical applications and methods of synthesis of hydroxyapatite.

If we observe the bone at the microscopic level, we find the cellular composition of osteocytes and the matrix. A matrix composed of collagen fibers helps to give strength and flexibility to the bone and is associated with HAp microcrystals and mineral salts for hardness. The bone tissue is constantly replaced and remodeled, leading to the name Bone Remodeling. The stimulus from the osteocytes supports to initiate osteoclast and osteoblast for remodeling [110]. Bioactive ceramics act as an HAp layer on the fractured part, help to rise the osteoblast quickly to heal bone. Furthermore, studies proposed a combination of the HA layer with high-density polyethylene (HDPE) as a auxiliary material for bone [111,112,113,114,115]. This has been tested and commercially named HAPEXTM. Previously provided empirical results for osteoblast over a bioactive ceramic layer of HAp particles [112]. Composites from the bioactive ceramics joint with HAp particles and collagen fibres have possibility to act as an artificial substitute for bone [116]. The established graft have excellent mechanical properties [117]. HAp/alumina has been proposed as a bone substitute [118]. One study also concludes that mixing P_2_O_5_ glass with HAp achieves close to natural bone properties [119].

## 7. Hyaluronic Acid-Based Nanoparticles

**Figure 8:**
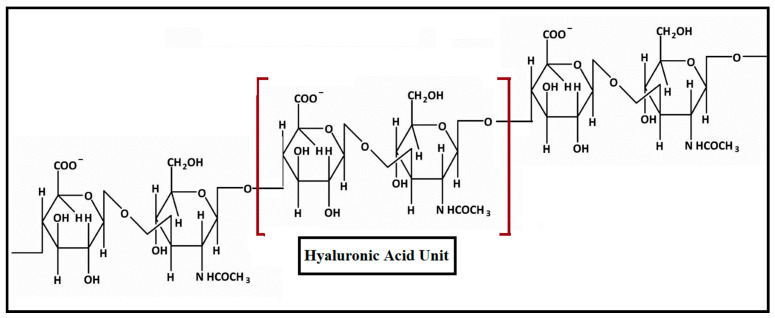
The structure of hyaluronic acid.

Hyaluronic acid (HA) is an anionic polysaccharide with repeating units of N-acetyl-D glucosamine and disaccharides of D-glucuronic acid linked via β-1,4- or β-1,3-glycosidic bonds [121,122]. HA also is known as hyaluronan, present in the synovial fluids, in the extracellular matrix, in the skin and uniformly distributed in vertebrate tissues of the body [123]; the structure is shown in Figure 8. HA has good biocompatibility, greater viscoelasticity, biodegradability and the capacity to combine with the receptor cell surface. The presence of receptors such as CD44 on the surface of HA makes it a site-specific drug delivery system for anticancer drug release and its capacity for cellular uptake [124,125].

Modification of HA with hydrophobic macromolecules occurs because of hydroxyl and carboxylic functional groups and its negatively charged surface. Based on nanocarriers, HA can be divided into different drug delivery systems such as gel and cationic drug delivery systems, a polyelectrolyte microcapsule release system, a nano-emulsion delivery system, and a nano-carriers drug delivery system [126]. HA nanocarriers are not only used for cancer drug therapy but also as photosensors, for photo imaging, and gene plasmids [127]. Various modification steps can be used to enhance its properties for drug delivery systems, such as conjugation with the nanocarriers as a targeting moiety, with dendrimers, quantum dots, and graphene oxide [35,128]. Modification of HA is important due to its accumulation in the liver; hence, Choi et al. [129] synthesized polyethylene glycol-modified HA nanoparticles. By varying the degree of PEGlation, negatively charged self-assembled nanoparticles were formed. Although PEGlation of HA-NPs decreased their cellular uptake in vitro, nanoparticles having CD44HA receptor were taken in huge amount by cancer cell as compared to normal fibroblast cells. Using in vivo images, it was established that PEGlation of HA reduces the accretion of HA nanoparticles in the liver and enhances its cellular uptake. Characterization results showed the maximum accumulation of PEGylated HA nanoparticles in tumour cells. Lee and Na [130] also synthesized the sustainable drug release to reduce the toxic effect and avoid an accumulation of HA in healthy organs. The authors modified HA with a Polycaprolacton (PCL) copolymer, HA cross-linking is carried out by disulfide bond. Doxorubicin (DOX), selected as a sample anticancer drug, was successfully encapsulated into the nanoparticles with greater drug loading ability. The DOX-loaded in the modified HA significantly delayed the drug release in physiological environments. However, the drug release rate was significantly improved in the occurrence of glutathione, a thiol-containing tripeptide capable of reducing disulfide bonds in the cytoplasm. Moreover, DOX-HA-ss-NPs could efficiently transport the DOX into the tumour cell. Overall, the characterization results indicate that this modification can act as a potential carrier for a drug delivery system. In another study [131], photo-crosslinked hyaluronic acid nanoparticles (c-HANPs) were prepared for involuntary burst release of the drug into the blood. They were readily synthesized via UV-triggered chemical cross-linking with the acrylate groups in the polymer backbone. High sustainability of c-HANPs enabled their large circulation in the body. Owing to the constant discharge of the drug and improved tumour-targeting capacity, c-HANPs showed higher healing ability compared to uncrosslinked HANPs. These data imply the promising potential of c-HANP as a tumour-targeting drug carrier and have established the extraordinary effect of better stability upon the biodistribution and therapeutic ability of drug-loaded nanoparticles.

## 8. Conclusions

In this review, five natural biocompatible and biodegradable polymer-based nanoparticles have been critically examined for therapeutic applications. The nanoformulations are superior to macro or conventional drug delivery systems, and they can maximize dosing frequency. They can target infected areas, organs, tumor sites, and tissues in the body. Biodegradable and biocompatible polymers are appropriate materials for the development of novel drug delivery systems. Biocompatibility, mechanical properties, and low cytotoxic effects of these polymers make them an appropriate choice for drug delivery systems. It is the need of time to manipulate the system, which reduces the toxic effects of drugs on healthy organs or body parts. Still, there is room to explore more biodegradable polymers for innovative biomedical applications. With increased progress in nanotechnology, we expect progress in the area of therapeutic systems based on the development of modified nanomedicines for the proper treatment of diseases.

## Figures and Tables

**Table 1:** Some ideal properties of polymeric nanocarriers [3].

Nanoparticle Drug Delivery System
Simple and low-cost to synthesize and scale-up
High shear forces, no heat or organic solvents involved in their preparation process
Stable and reproducible
Appropriate for a broad classification of drugs: proteins, small molecules, and polynucleotides
Capacity to lyophilize
Stable after synthesis
**Polymeric carriers**
Non-toxic
Biocompatible
Non-immunogenic
Water-soluble
Inexpensive
Easy to synthesize and characterize

**Table 2:** Shows the modified chitosan-, alginate-, and albumin-based nano polymer for drug delivery systems [8].

Polymer/Modified Polymer	Preparation Method	Model Drug
Chitosan–folic acid	Ionic gelation	5-flurouracil
Chitosan/alginate and solid lipid NPs	Ionic gelation	Ciprofloxacin
PVA/chitosan-gelation	Electrospinning method	Erythromycin
Alginate calcium carbonate	Co-precipitation	Paclitaxel
PEGylated albumin	RAFT polymerization	Lysozyme
Bovine serum albumin	Multistep process	Temozolomide
Egg albumin	Desolvation method	Curcumin
PEG-modified human serum albumin	Solid dispersion technology	Paclitaxel

**Table 3:** Shows the advantages, disadvantages, biomedical applications, and methods of preparation for hydroxyapatite [120].

Advantages	Disadvantages	Biomedical Applications	Method of Preparation
Bioactive (hydration shell)	Strong hydration shell, ionic surface, fragile	Drug delivery system, tissue engineering’ implants	Wet method, dry method, high-temperature process
Ease of modification and surface functionalization	Precipitation and turbid solution	-	-
Good attachment to polymers	Surface corona formation, aggregation	-	-
Ease of composite formation	Dispersity in chemical composition, size and shape polymorphism	-	-
Biodegradable, biocompatibility	High pH sensitivity and solubility	-	-
Self-assembly	Low stability	-	-

## References

[1] Sur S., Rathore A., Dave V., Reddy K.R., Chouhan R.S., Sadhu V. (2019). Recent developments in functionalized polymer nanoparticles for efficient drug delivery system. Nano Struct. Nano Objects.

[2] Tong X., Pan W., Su T., Zhang M., Domg W., Qi X. (2020). Recent advances in natural polymer-based drug delivery systems. Reactive and Functional Polymers. React. Funct. Polym..

[3] Tiyaboonchai W. (2003). Chitosan Nanoparticles: A Promising System for Drug Delivery. Naresuan Univ. J..

[4] Jeong B., Choi Y.K., Bae Y.H., Zentner G., Kim S.W. (1999). New biodegradable polymers for injectable drug delivery systems. J. Control. Release.

[5] Kumari A., Yadav S.K., Yadav S.C. (2010). Biodegradable polymeric nanoparticles based drug delivery systems. Colloids Surf. B Biointerfaces.

[6] Marianecci C., Di Marzio L., Rinaldi F., Celia C., Paolino D., Alhaique F., Esposito S., Carafa M. (2014). Niosomes from 80s to present: The state of the art. Adv. Colloid Interface Sci..

[7] Tibbitt M.W., Dahlman J.E., Langer R. (2016). Emerging frontiers in drug delivery. J. Am. Chem. Soc..

[8] George A., Shah P.A., Shrivastav P.S. (2019). Natural biodegradable polymers based nano-formulations for drug delivery: A review. Int. J. Pharm..

[9] Hassan S., Zhang X. (2019). Droplet-Based Microgels: Attractive Materials for Drug Delivery Systems. Res. Dev. Mater. Sci..

[10] Ramkumar V.S., Pugazhendhi A., Gopalakrishnan K., Sivagurunathan P., Saratale G.D., Dung T.N.B., Kannapiran E. (2017). Biofabrication and characterization of silver nanoparticles using aqueous extract of seaweed Enteromorpha compressa and its biomedical properties. Biotechnol. Rep..

[11] Chen M., Yin M. (2014). Design and development of fluorescent nanostruc-tures for bioimaging. Prog. Polym. Sci..

[12] Baba M., Matsumoto Y., Kashio A., Cabral H., Nishiyama N., Kataoka K., Yamasoba T. (2012). Micellization of cisplatin (NC-6004) reduces its oto-toxicity in guinea pigs. J. Control. Release.

[13] Cramer N.B., Standsburry J.W., Bowman C.N. (2011). Recent advances and developments in composite dental restorative materials. J. Dent. Res..

[14] Mukherjee S., Vinugopal J.R., Ravichandran R., Ramalingam M., Raghunath M., Ramakrishna S. (2013). Nanofiber technology for controllingstem cell functions and tissue engineering. Micro Nanotechnol. Eng. Stem Cells Tissues.

[15] Cheng Z., Zaki A.A., Hui J.Z., Muzykantov V.R., Tsourkas A. (2012). Multifunctional nanoparticles: Cost versus benefit of adding targeting andimaging capabilities. Science.

[16] Shi J., Votruba A.R., Farokhzad O.C., Langer R. (2010). Nanotechnology in drug delivery and tissue engineering: From discovery to applications. Nano Lett..

[17] Liu W., Thomopoulos S., Xia Y. (2012). Electrospun nanofibers for regenera-tive medicine. Adv. Healthc. Mater..

[18] De Jong W.H., Borm P.J.A. (2008). Drug delivery and nanoparticles: Applications and hazards. Int. J. Nanomed..

[19] Pugazhendhi A., Prabakar D., Jacob J.M., Karuppusamy I., Saratale R.G. (2018). Synthesis and characterization of silver nanoparticles using Gelidium amansii and its antimicrobial property against various pathogenic bacteria. Microb. Pathog..

[20] Nanotechnology for Health: Vision Paper and Basis for a Strategic Agenda for Nanomedicine. https://op.europa.eu/en/publication-detail/-/publication/60816548-e372-4216-a253c4442b527a21/language-en.

[21] Kamaly N., Yameen B., Wu J., Farokhzad O.C. (2016). Degradable controlled-release polymers and polymeric nanoparticles: Mechanisms of controlling drug release. Chem. Rev..

[22] Soppimath K.S., Aminabhavi T.M., Kulkarni A.R., Rudzinski W.E. (2001). Biodegradable polymeric nanoparticles as drug delivery devices. J. Control. Release.

[23] Nejati-Koshki K., Mesgari M., Ebrahimi E., Abbasalizadeh F., Aval S.F., Khandaghi A.A., Abasi M., Akbarzadeh A. (2014). Synthesis and in vitro study of cisplatin-loaded Fe_3_O_4_ nanoparticles modified with PLGA-PEG_6000_ copolymers in treatment of lung cancer. J. Microencapsul..

[24] Conde J., Larguinno M., Cordeiro A., Raposo L.R., Costa P.M., Santos S., Diniz M.S., Fernandes A.R., Baptista P.V. (2013). Gold-nanobeacons for gene therapy: Evaluation of genotoxicity, cell toxicity, and proteome profiling analysis. Nanotoxicology.

[25] Brannon-Peppas L., Blanchette J.O. (2012). Nanoparticle and targeted systems for cancer therapy. Adv. Drug Deliv. Rev..

[26] Feng S.S. (2014). Nanoparticles of biodegradable polymers for new-concept chemotherapy. Expert Rev. Med. Devices.

[27] Yeo W.W.Y., Hosseinkhani H., Abdul Rahman S., Rosli R., Domb A.J., Abdullah S. (2014). Safety Profile of Dextran-Spermine Gene Delivery Vector in Mouse Lungs. J. Nanosci. Nanotechnol..

[28] Alibolandi M., Abnous K., Ramezani M., Hosseinkhani H., Hadizadeh F. (2014). Synthesis of AS1411-aptamer-conjugated CdTe quantum dots with high fluorescence strength for probe labeling tumor cells. J. Fluoresc..

[29] Dragan E.S., Bucataria F. (2016). Design and characterization of anionic hydrogels confined in Daisogel silica composites microspheres and their application in sustained release of proteins. Colloid. Surf. A.

[30] Farokhi M., Mottaghitalab F., Shokrgozar M.A., Ou K.L., Mao C., Hosseinkhani H. (2016). Importance of dual delivery systems for bone tissue engineering. J. Control. Release.

[31] Qi X., Wei W., Li J., Liu Y., Hu X., Zhang J., Bi L., Dong W. (2015). Fabrication and Characterization of a Novel Anticancer Drug Delivery System: Salecan/Poly(methacrylic acid) Semi-interpenetrating Polymer Network Hydrogel. ACS Biomater. Sci. Eng..

[32] Dinu M.V., Dragan E.S. (2010). Evaluation of Cu^2+^, Co^2+^ and Ni^2+^ ions removal from aqueous solution using a novel chitosan/clinoptilolite composite: Kinetics and isotherms. Chem. Eng. J..

[33] Qi X., Wei W., Li J., Zuo G., Pan X., Su T., Zhang J., Dong W. (2017). Salecan-Based pH-Sensitive Hydrogels for Insulin Delivery. Mol. Pharmaceut..

[34] Pande V. (2015). Studies on the characteristics of zaltoprofen loaded gelatin nanoparticles by nanoprecipitation. Inventi Rapid NDDS.

[35] Prajapati S.K., Jain A., Jain A. (2019). Biodegradable polymers and constructs: A novel approach in drug delivery. Eur. Polym. J..

[36] Ossipov D.A. (2010). Nanostructured hyaluronic acid-based materials for active delivery to cancer. Expert Opin. Drug Deliv..

[37] Kluin O.S., van der Mei H.C., Busscher H.J., Neut D. (2013). Biodegradable vs. non-biodegradable antibiotic delivery devices in the treatment of osteomyelitis. Expert Opin. Drug Deliv..

[38] Shafabakhsh R., Yousefi B., Asemi Z., Nikfar B., Mansournia M.A., Hallajzadeh J. (2020). Chitosan: A compound for drug delivery system in gastric cancer—A review. Carbohydr. Polym..

[39] LeHoux J.G., Grondin F. (1993). Some effects of chitosan on liver function in the rat. Endocrinology.

[40] Peniston Q.P., Johnson E.L. (1980). Process for the Manufacturer of Chitosan. U.S. Patent.

[41] Bernkop-Schnürch A., Dünnhaupt S. (2012). Chitosan-based drug delivery systems. Eur. J. Pharm. Biopharm..

[42] Huang G., Liu Y., Chen L. (2017). Chitosan and its derivatives as vehicles for drug delivery. Drug Deliv..

[43] Prabaharan M., Mano J.F. (2005). Chitosan-Based Particles as Controlled Drug Delivery Systems. Drug Deliv..

[44] Maitra A., Kumar P.G., De T., Sahoo S.K. (1999). Process for the Preparation of Highly Monodispersed Hydrophilicpolymeric Nanoparticles of Size Less than 100 nm. U.S. Patent.

[45] Calvo P., Remunan-Lopez C., Vila-Jato J.L., Alonso M.J. (1997). Chitosan and chitosan/ethylene oxide propylene oxide block copolymer nanoparticles as novel carriers for proteins and vaccines. Pharm. Res..

[46] Janes K.A., Fresneau M.P., Marazuela A., Fabra A., Alonso M.J. (2001). Chitosan nanoparticles as delivery systems for doxorubicin. J. Control. Release.

[47] Pan Y., Li Y.J., Zhao H.Y., Zheng J.M., Xu H., Wei G., Hao J.S., Cui F.D. (2002). Bioadhesive polysaccharide in protein delivery system: Chitosan nanoparticles improve the intestinal absorption of insulin in vivo. Int. J. Pharm..

[48] Giacone D.V., Dartora V.F., de Matos J.K., Passos J.S., Miranda D.A., de Oliveira E.A., Silveira E.R., Costa-Lotufo L.V., Maria-Engler S.S., Lopes L.B. (2020). Effect of nanoemulsion modification with chitosan and sodium alginate on the topical delivery and efficacy of the cytotoxic agent piplartine in 2D and 3D skin cancer models. Int. J. Biol. Macromol..

[49] El-Shabouri M.H. (2002). Positively charged nanoparticles for improving the oral bioavailability of cyclosporin-A. J. Pharm..

[50] Bodmeier R., Chen H.G., Paeratakul O. (1989). A novel approach to the oral delivery of micro- or nanoparticles. Pharm. Res..

[51] Afshar M., Dai Y.-N., Zhang J.-P., Wang A.-Q., Wei Q. (2020). Preparation and characterization of sodium alginate/polyvinyl alcohol hydrogel containing drug-loaded chitosan nanoparticles as a drug delivery system. J. Drug Deliv. Sci. Technol..

[52] Gomathi T., Sudha P.N., Florence J.A.K., Venkatesan J., Anil S. (2017). Fabrication of letrozole formulation using chitosan nanoparticles through ionic gelation method. Int. J. Biol. Macromol..

[53] Wang J., Tan J., Luo J., Huang P., Zhou W., Chen L., Long L., Zhang L.M., Zhu B., Yang L. (2017). Enhancement of scutellarin oral delivery efficacy by vitamin B12-modified amphiphilic chitosan derivatives to treat type II diabetes induced-retinopathy. J. Nanobiotechnol..

[54] Zhang Y., Lv T., Zhang H., Xie X., Li Z., Chen H. (2017). Folate and heptamethine cyanine modified chitosan-based nanotheranostics for tumor targeted near-infrared fluorescence imaging and photodynamic therapy. Biomacromolecules.

[55] Kamel K., Khalil I.A., Rateb M.E., Elgendy H., Elhawary S. (2017). Chitosan-coated cinnamon/oregano-loaded solid lipid nanoparticles to augment 5-fluorouracil cytotoxicity for colorectal cancer: Extracts standardization, nanoparticles optimization and cytotoxicity evaluation. J. Agric. Food Chem..

[56] Lin J.T., Liu Z.K., Zhu Q.L., Rong X.H., Liang C.L., Wang J. (2017). Redox-responsive nano-carriers for drug and gene co-delivery based on chitosan derivatives modified mesoporous silica nanoparticles. Colloids Surf. B Biointerfaces.

[57] Gu J., Bayati K., Ho E.A. (2017). Development of antibody-modified chitosan nanoparticles for the targeted delivery of siRNA across the blood-brain barrier as a strategy for inhibiting HIV replication in astrocytes. Drug Deliv. Transl. Res..

[58] Park J.H., Kwon S., Lee M., Chung H., Kim J.H., Kim Y.S., Park R.W., Kim I.S., Seo S.B., Kwon I.C. (2006). Self-assembled nanoparticles based on glycol chitosan bearing hydrophobic moieties as carriers for doxorubicin: In vivo biodistribution and anti-tumor activity. Biomaterials.

[59] Belbekhouche S., Mansour O., Carbonnier B. (2018). Promising sub-100 nm tailor made hollow chitosan/poly(acrylic acid) nanocapsules for antibiotic therapy. J. Colloid Interface Sci..

[60] Calvo P., Remunan C., Jato J.L.V., Alonso M.J. (1997). Novel hydrophilic chitosan-polyethylene oxide nanoparticles as protein carriers. J. Appl. Polym.Sci..

[61] Shu X.Z., Zhu K.J. (2002). The influence of multivalent phosphate structure on the properties of ionically cross-linked chitosan films for controlled drug release. Eur. J. Pharm. Biopharm..

[62] Peng Y., Guo J.J., Liu Y.M., Wu X.L. (2014). MicroRNA-34A inhibits the growth, invasion and metastasis of gastric cancer by targeting PDGFR and MET expression. Biosci. Rep..

[63] Chi J., Jiang Z., Qiao J., Zhang W., Peng Y., Liu W., Han B. (2019). Antitumor evaluation of carboxymethyl chitosan based norcantharidin conjugates against gastric cancer as novel polymer therapeutics. Int. J. Biol. Macromol..

[64] Gong Y., Tao L., Wang F., Liu W., Jing L., Liu D., Zhou N. (2015). Chitosan as an adjuvant for a Helicobacter pylori therapeutic vaccine. Mol. Med. Rep..

[65] Arora S., Aggarwal P., Pathak A., Bhandari R., Duffoo F., Gulati S.C. (2012). Molecular genetics of head and neck cancer (review). Mol. Med. Rep..

[66] Øilo M., Bakken V. (2015). Biofilm and dental biomaterials. Materials.

[67] Donlan R.M. (2002). Biofilms: Microbial life on surfaces. Emerg. Infect. Dis..

[68] Schiroky L.V., Garcia I.M., Ogliari F.A., Samuel S.M.W., Collares F.M. (2017). Triazine compound as copolymerized antibacterial agent in adhesive resins. Braz. Dent. J..

[69] Degrazia F.W., Leitune V.C.B., Gracia I.M., Arthur R.A., Samuel S.M.W., Collares F.M. (2016). Effect of silver nanoparticles on the physicochemical and antimicrobial properties of an orthodontic adhesive. J. Appl. Oral. Sci..

[70] Wassel M.O., Khattab M.A. (2017). Antibacterial activity against Streptococcus mutans and inhibition of bacterial induced enamel demineralization of propolis, miswak, and chitosan nanoparticles based dental varnishes. J. Adv. Res..

[71] Zhang N., Chen C., Melo M.A., Bai Y., Cheng L., Xu H.H. (2015). A novel protein-repellent dental composite containing 2-methacryloyloxyethyl phosphorylcholine. Int. J. Oral. Sci..

[72] Fornell A.C., Skold-Larsoon K., Hallgren A., Bergstand F., Twetman S. (2002). Effect of a hydrophobic tooth coating on gingival health, mutans streptococci, and enamel demineralization in adolescents with fixed orthodontic appliances. Acta Odontol. Scand..

[73] Nguyen S., Hiorth M., Rykke M., Smistad G. (2013). Polymer coated liposomes for dental drug delivery—Interactions with parotid saliva and dental enamel. Eur. J. Pharm. Sci..

[74] Tonnesen H.H., Karlsen J. (2002). Alginate in Drug Delivery Systems. Drug Dev. Ind. Pharm..

[75] Daemi H., Barikani M. (2012). Synthesis and characterization of calcium alginate nanoparticles, sodium homopolymannuronate salt and its calcium nanoparticles. Sci. Iran..

[76] Yang J.-S., Xie Y.-J., He W. (2011). Research progress on chemical modification of alginate: A review. Carbohydr. Polym..

[77] Ciofani G., Raffa V., Pizzorusso T., Menciassi A., Dario P. (2008). Characterization of an alginate-based drug delivery system for neurological applications. Med. Eng. Phys..

[78] Lencina M.S., Ciolino A.E., Andreucetti N.A., Villar M.A. (2015). Thermoresponsive hydrogels based on alginate-g-poly (N-isopropylacrylamide) copolymers obtained by low doses of gamma radiation. Eur. Polym. J..

[79] Gao X., Yu Z., Liu B., Yang J., Yang X., Yu Y. (2020). A smart drug delivery system responsive to pH/enzyme stimuli based on hydrophobic modified sodium alginate. Eur. Polym. J..

[80] Binauld S., Stenzel M. (2013). Acid-degradable polymers for drug delivery: A decade of innovation. Chem. Commun..

[81] Liu M., Song X., Wen Y., Zhu J.-L. (2017). Injectable thermoresponsive hydrogel formed by alginate-g-poly (N-isopropylacrylamide) that releases doxorubicin-encapsulated micelles as a smart drug delivery system. ACS Appl. Mater. Interfaces.

[82] Luo R.C., Lim Z.H., Li W., Shi P., Chen C.-H. (2014). Near-infrared light triggerable deformation-free polysaccharide double network hydrogels. Chem. Commun..

[83] Dong L., Xia S., Wu K., Huang Z., Chen H., Chen J., Zhang J. (2010). A pH/enzyme-responsive tumor-specific delivery system for doxorubicin. Biomaterials.

[84] Wu J.L., Wang C.Q., Zhuo R.X., Cheng S.X. (2014). Multi-drug delivery system based on alginate/calcium carbonate hybrid nanoparticles for combination chemotherapy. Colloids Surf. B Biointerfaces.

[85] Zhang B., Yan Y., Shen Q., Ma D., Huang L., Cai X., Tan S. (2017). A colon targeted drug delivery system based on alginate modificated graphene oxide for colorectal liver metastasis. Mater. Sci. Eng. C.

[86] Nazemi Z., Nourbakhsh M.S., Kiani S., Daemi H., Ashtiani M.K., Baharv H. (2020). Effect of chemical composition and sulfated modification of alginate in the development of delivery systems based on electrostatic interactions for small molecule drugs. Mater. Lett..

[87] Hügl S., Scheper V., Gepp M.M., Lenarz T., Rau T.S., Schwieger J. (2019). Coating stability and insertion forces of an alginate-cell-based drug delivery implant system for the inner ear. J. Mech. Behav. Biomed. Mater..

[88] Rahimnejad M., Jahanshahi M., Najafpour G.D. (2006). Production of biological nanoparticles from bovine serum albumin for drug delivery. Afr. J. Biotechnol..

[89] Joshi M., Nagarsenkar M., Prabhakar B. (2020). Albumin nano-carriers for pulmonary drug delivery: An attractive approach. J. Drug Deliv. Sci. Technol..

[90] Patil G.V. (2003). Biopolymer albumin for diagnosis and in drug delivery. Drug Dev. Res..

[91] Irache J.M., Merodio M., Arnedo A., Camapanero M.A., Mirshahi M., Espuelas S. (2005). Albumin nanoparticles for the intravitreal delivery of anticytomegaloviral drugs. Mini Rev. Med. Chem..

[92] Arshady R. (1989). Preparation of microspheres and microcapsules by interfacial polycondensation techniques. J. Microcapsul..

[93] Oakenfull D., Pearce J., Burley R.W., Damodaran S., Paraf A. (1997). Food Proteins and Their Applications.

[94] Hu Y.J., Liu Y., Sun T.Q., Bai A.M., Lü J.Q., Pi Z.B. (2006). Binding of anti-inflammatory drug cromolyn sodiumto bovine serumalbumin. Int. J. Biol. Macromol..

[95] Kratz F. (2008). Albumin as a drug carrier: Design of prodrugs, drug conjugates and nanoparticles. J. Control. Release.

[96] Ulbrich K., Hematara T., Herbert E., Kreuter J. (2009). Transferrin- and transferrinreceptor-antibody-modified nanoparticles enable drug delivery across the blood-brain barrier (BBB). Eur. J. Pharm. Biopharm..

[97] Elzoghby A.O., Samy W.M., Elgindy N.A. (2012). Albumin-based nanoparticles as potential controlled release drug delivery systems. J. Control. Release.

[98] Kim T.H., Jiang H.H., Youn Y.S., Park C.W., Tak K.K., Lee S., Kim H., Jon S., Chen X., Lee K.C. (2011). Preparation and characterization of water-soluble albuminbound curcumin nanoparticles with improved antitumor activity. Int. J. Pharm..

[99] Dreis S., Rothweiler F., Michaelis M., Cinatl J., Kreuter J., Langer K. (2007). Preparation, characterization and maintenance of drug efficacy of doxorubicin-loaded human serum albumin (HSA) nanoparticles. Int. J. Pharm..

[100] Iwao Y., Tomiguchi I., Domura A., Mantaira Y., Minami A., Suzuki T., Ikawa T., Kimura S.I., Itai S. (2018). Inflamed site-specific drug delivery system based on the interaction of human serum albumin nanoparticles with myeloperoxidase in a murine model of experimental colitis. Eur. J. Pharm. Biopharm..

[101] Siri M., Grasselli M., Alonso S.d.V. (2020). Correlation between assembly structure of a gamma irradiated albumin nanoparticle and its function as a drug delivery system. Colloids Surf. A Physicochem. Eng. Asp..

[102] Stein N., Mulac D., Fabian J., Herrmann F.C., Langer K. (2020). Nanoparticle albumin-bound mTHPC for photodynamic therapy: Preparation and comprehensive characterization of a promising drug delivery system. Int. J. Pharm..

[103] Gomes D.S., Santos A.M.C., Neves G.A., Menezes R.R. (2019). A brief review on hydroxyapatite production and use in biomedicine. Cerâmica.

[104] Zhao Z., Espanol M., Guillem-Marti J., Kempf D., Diez-Escudero A., Ginebra M.-P. (2016). Ion-doping as a strategy to modulate hydroxyapatite nanopartilce internalization. Nanoscale.

[105] Cellet T.S.P., Pereira G.M., Muniz E.C., Silva R., Rubira A.F. (2015). Hydroxyapatite nanowhiskers embedded in chondroitin sulfate microsphere as colon targeted drug delivery system. J. Mater. Chem. B.

[106] Sionkowska A., Kozlowsa J. (2010). Characterization of collagen/hydroxyapatite composite sponges as a potential bone substitute. Int. J. Biol. Macromol..

[107] Li D., Huang X., Wu Y., Li J., Cheng W., He J., Tian H., Huang Y. (2016). Preparation of pH-responsive mesoporous hydroxyapatite nanoparticles for intracellular controlled release of an anticancer drug. Biomater. Sci..

[108] Venkatasubbu G.D., Ramasamy S., Avadhani G.S., Ramakrishnan V., Kumar J. (2013). Surface modification and paclitaxel drug delivery of folic acid modified polyethylene glycol functionalized hydroxyapatite nanoparticles. Powder Technol..

[109] Ghafarinazari A., Tahari A., Moztarzadeh F., Mozafari M., Bahroloom M.E. (2011). Ion exchange behaviour of silver-doped apatite micro and nanoparticles as antibacterial biomaterial. Micro Nano Lett..

[110] Mizuno S., Glowacki J. (1996). Three dimensional composite of demineralized bone powder and collagen for in vitro analysis of chondroinduction of human dermal fibroblasts. Biomaterials.

[111] Ramakrishna S., Mayer J., Wintermantel E., Leong K.W. (2001). Biomedical applications of polymer-composite materials: A review. Compos. Sci. Technol..

[112] Wang M., Joseph R., Bonfield W. (1998). Hydroxyapatite-poly- ethylene composites for bone substitution: Effects of ceramic particle size and morphology. Biomaterials.

[113] Bonfield W., Wang M., Tanner K.E. (1998). Interfaces in analogue biomaterials. Acta Mater..

[114] Di Silvio L., Dalby M.J., Bonfield W. (2002). Osteoblast behaviour on HA/PE composite surfaces with different HA volumes. Biomaterials.

[115] Wang M., Bonfield W. (2000). Chemically coupled hydroxyapatite-polyethylene composites: Processing and characterization. Mater. Lett..

[116] Kikuchi M., Itoh S., Ichinose S., Shinomiya K., Tanaka J. (2001). Self-organization mechanism in a bone-like hy-droxyapatite/collagen nanocomposite synthesized in vitro and its biological reaction in vivo. Biomaterials.

[117] Li J., Fartash B., Hermannson L. (1995). Hydroxyapatite-alu-mina composites and bone-bonding. Biomaterials.

[118] Lopes M.A., Silva R.F., Monteiro F.J., Santos J.D. (2000). Microstructural dependence of Young’s moduli of P_2_O_5_ glass reinforced hydroxyapatite for biomedical applications. Biomaterials.

[119] Liu Q., de Wijn J.R., van Blitterswijk C.A. (1998). Composite biomaterials with chemical bonding between hy-droxyapatite filler particles and PEG/PBT copoly-mer matrix. J. Biomed. Mater. Res..

[120] Ghiasi B., Sefidbakht Y., Rezaei M. (2019). Hydroxyapatite for Biomedicine and Drug Delivery. Nanomater. Adv. Biol. Appl..

[121] Choi K.Y., Min K.H., Na J.H., Choi K., Kim K., Park J.H., Kwon I.C., Jeong S.Y. (2009). Self-assembled hyaluronic acid nanoparticles as a potential drug carrier for cancer therapy: Synthesis, characterization, and in vivo biodistribution. J. Mater. Chem..

[122] Schanté C.E., Zuber G., Herlin C., Vandamme T.F. (2011). Chemical modifications of hyaluronic acid for the synthesis of derivatives for a broad range of biomedical applications. Carbohydr. Polym..

[123] Cheng D., Han W., Yang K., Song Y., Jiang M., Song E. (2014). One-step facile synthesis of hyaluronic acid functionalized fluorescent gold nanoprobes sensitive to hyaluronidase in urine specimen from bladder cancer patients. Talanta.

[124] Liu K., Wang Z.Q., Wang S.J., Liu P., Qin Y.H., Ma Y., Li X.C., Huo Z.-J. (2015). Hyaluronic acidtagged silica nanoparticles in colon cancer therapy: Therapeutic efficacy evaluation. Int. J. Nanomed..

[125] Wang H., Sun G., Zhang Z., Ou Y. (2017). Transcription activator, hyaluronic acid and tocopheryl succinate multi-functionalized novel lipid carriers encapsulating etoposide for lymphoma therapy. Biomed. Pharmacother..

[126] Huang G., Huang H. (2018). Hyaluronic acid-based biopharmaceutical delivery and tumor-targeted drug delivery system. J. Control. Release.

[127] Zhong W., Pang L., Feng H., Dong H., Wang S., Cong H., Shen Y., Bing Y. (2020). Recent advantage of hyaluronic acid for anticancer application: A review of “3S” transition approach. Carbohydr. Polym..

[128] Cai J., Fu J., Li R., Zhang F., Ling G., Zhang P. (2019). A potential carrier for anti-tumor targeted delivery-hyaluronic acid nanoparticles. Carbohydr. Polym..

[129] Choi K.Y., Min K.H., Yoon H.Y., Kim K., Park J.H., Kwon I.C., Choi K., Jeong S.Y. (2011). PEGylation of hyaluronic acid nanoparticles improves tumor targetability in vivo. Biomaterials.

[130] Na K., Lee C.-S. (2014). Photochemically Triggered Cytosolic Drug Delivery Using pHResponsive Hyaluronic Acid Nanoparticles for Light-Induced Cancer Therapy. Biomaterials.

[131] Yoon H.Y., Koo H., Ki Choi Y., Kwon I.C., Choi K., Jae HyungPark J.H., Kim K. (2013). Photo-crosslinked hyaluronic acid nanoparticles with improved stability for in vivo tumor-targeted drug delivery. Biomaterials.

